# Effects of Tebuconazole Application on Soil Microbiota and Enzymes

**DOI:** 10.3390/molecules27217501

**Published:** 2022-11-03

**Authors:** Małgorzata Baćmaga, Jadwiga Wyszkowska, Agata Borowik, Jan Kucharski

**Affiliations:** Department of Soil Science and Microbiology, Faculty of Agriculture and Forestry, University of Warmia and Mazury in Olsztyn, Plac Łódzki 3, 10-727 Olsztyn, Poland

**Keywords:** tebuconazole, soil microbiome, genetic diversity, biochemical activity of soil

## Abstract

Identification of pesticide impact on the soil microbiome is of the utmost significance today. Diagnosing the response of bacteria to tebuconazole, used for plant protection, may help isolate the most active bacteria applicable in the bioaugmentation of soils contaminated with this preparation. Bearing in mind the above, a study was undertaken to test the effect of tebuconazole on the diversity of bacteria at all taxonomic levels and on the activity of soil enzymes. It was conducted by means of standard and metagenomic methods. Its results showed that tebuconazole applied in doses falling within the ranges of good agricultural practice did not significantly disturb the biological homeostasis of soil and did not diminish its fertility. Tebuconazole was found to stimulate the proliferation of organotrophic bacteria and fungi, and also the activities of soil enzymes responsible for phosphorus, sulfur, and carbon metabolism. It did not impair the activity of urease responsible for urea hydrolysis, or cause any significant changes in the structure of bacterial communities. All analyzed soil samples were mainly populated by bacteria from the phylum *Proteobacteria*, *Actinobacteria*, *Firmicutes*, *Gemmatimonadetes*, *Acidobacteria*, *Planctomycetes*, and *Chloroflexi*. Bacteria from the genera *Kaistobacter*, *Arthrobacter*, and *Streptomyces* predominated in the soils contaminated with tebuconazole, whereas these from the *Gemmata* genus were inactivated by this preparation.

## 1. Introduction

The impact of anthropogenic activity on the natural environment bears the risk of hindering the proper functioning of various ecosystems, including (in particular) the soil ecosystem, being a fundament of the agriculture. Soil dysfunction triggered by, inter alia, drought, shortage of nutrients, pollution with various compounds, and salination, may significantly affect food safety [1]. The intensification of agriculture, entailing pesticide use, may adversely influence both the biodiversity and functioning of soil ecosystems [2,3]. These unbeneficial changes proceeding in soil may also contribute to the impaired development of crops, which most often leads to deterioration of the quality and quantity of their yield [4,5]. Therefore, continuous monitoring and assessment of the effects of chemicals used in agricultural production are essential, as their inconsiderate application may trigger severe changes in the soil environment [6,7,8]. Soil is deemed a non-renewable natural resource; hence, its appropriate quality and fertility underlie natural environment functioning [9]. One of the key factors ensuring the maintenance of soil quality and health is microorganisms. Being involved in the transformation of chemical compounds, they not only contribute to providing available nutrients to plants but also serve as promoters of their growth and development [6]. In addition, they secrete enzymes which either occur freely or are stabilized by organic matter in the soil environment. The soil enzymes are actively involved in biochemical processes in the soil; they are essential for the proper functioning of microorganisms by increasing the rate of reactions leading to organic matter degradation and release of nutrients to the soil [8,10,11]. A reliable marker of the functioning of soil exposed to pesticide effects is also the structure of communities of microorganisms involved in the interactions with other organisms and biological processes [12,13,14,15]. Even though fungicides positively affect the stabilization and improvement of agricultural productivity, their excessive and irrational use may lead to environmental contamination and eradication of non-target organisms. This, in turn, raises serious concerns over human and animal health [16]. Tebuconazole is an active substance of fungicides from the largest and the most commonly applied in agriculture group of triazoles. They have been introduced into plant protection by the Bayer company since 1973 [17]. Ever since, triazoles have become the most commonly applied group of fungicides, with tebuconazole introduced onto the market in 1986 being one of their major representatives [18]. Tebuconazole served not only for crop protection against fungal pathogens, but also for the protection of green areas [19]. By inhibiting the activity of lanosterol 14α-demethylase, tebuconazole diminishes the biosynthesis of ergosterol being the major constituent of cellular membranes of fungi [20]. The half-life of tebuconazole varies from 49 to 610 days. It is characterized by no, or very limited, mineralization of the triazole or chlorophenyl ring [21]. Its degradation in soil is influenced by various factors, including: soil pH, organic carbon content, soil biological properties (mainly activity, diversity and distribution of microorganisms), and the availability of an organic substrate for tebuconazole-degrading microorganisms. Apart from these factors, tebuconazole degradation is significantly affected by the environment temperature, soil moisture content, and the properties of the chemical itself [22]. Its accumulation in soil may pose a threat to soil ecosystems, surface and groundwater, as well as to soil and aquatic organisms. In addition, it is classified as strongly carcinogenic to man [20,23], toxic to the liver, inducing disorders of the endocrine system as well as triggering developmental and reproductive dysfunctions [24]. Given its toxicity and stability, development of the method for its elimination from the natural environment seems to be a priority. Lović et al. [20] reported *Enterobacter sakazakii* and *Serratia* sp. strains to exhibit tebuconazole-degrading capability, most likely due to their high tolerance to tebuconazole and to the fact that they possess appropriate genes, i.e.,: *opd* and *mpd*. Other authors [16] observed that a bacterial consortium composed of the following strains: *Pseudomonas putida* sp. B1, *Acinetobacter* sp. B2, and *Arthrobacter* sp. B3, degraded 93% of a tebuconazole + fenhexamid mixture within 30 days of incubation.

In order to gather exhaustive information about the effects of tebuconazole on changes in the structure and activity of communities of microorganisms, a study was undertaken to evaluate its effect on soil microorganisms and enzymes. Determination of the diversity and structure of bacteria enabled identifying and characterizing active bacterial taxa, thereby allowed achieving a complete picture of the structure of bacterial communities in the soil at all taxonomic levels.

## 2. Results

### 2.1. Response of Soil Microorganisms to Tebuconazole

The present study demonstrated a stimulating effect of tebuconazole on the population numbers of organotrophic bacteria and fungi. Its highest dose (T4) applied to the soil caused a 1.6-fold increase in the count of organotrophic bacteria and a 3.6-fold increase in the count of fungi. The population number of actinobacteria was positively affected by T1 dose and negatively affected by T2–T4 doses of the studied fungicide (Table S1).

Values of the colony development (CD) index of microorganisms were also diversified by tebuconazole doses (Table 1). The CD value of organotrophic bacteria was the highest in T2 soil (CD = 55.236), that of actinobacteria in T1 soil (CD = 47.087), and that of fungi in T4 soil (CD = 42.059). The highest mean CD value was determined for organotrophic bacteria (CD = 48.453), followed by fungi (CD = 36.315) and actinobacteria (CD = 28.543). 

Unlike actinobacteria, organotrophic bacteria and fungi proliferated the fastest in all soil types in the first days of incubation (Figure S1). In the first two days, the greatest increase in the population number of organotrophic bacteria was noted in T4 soil (K_s_ = 63.02%). The number of colonies of organotrophic bacteria and fungi increased until day 8 of incubation since soil suspension sowing on plates, whereas that of actinobacteria increased till day 10. 

The ecophysiological diversity of the tested microorganisms in soil was in part disturbed by tebuconazole (Table 2). In the case of organotrophic bacteria, the highest value of the EP index was noted in T4 soil, with the lowest one in T1 soil. These results are, however, inexplicit as tebuconazole diminished the ecophysiological diversity of organotrophic bacteria in T1 and T3 soils and did not modify it in T2 soil. The weakest effect of tebuconazole was observed in the case of actinobacteria because it decreased their EP only in T1 soil, while their EP values noted in T2–T4 soils were similar to those determined in C soil. In the case of fungi, it decreased their ecophysiological diversity only in T3 and T4 soils. 

**Figure 1:**
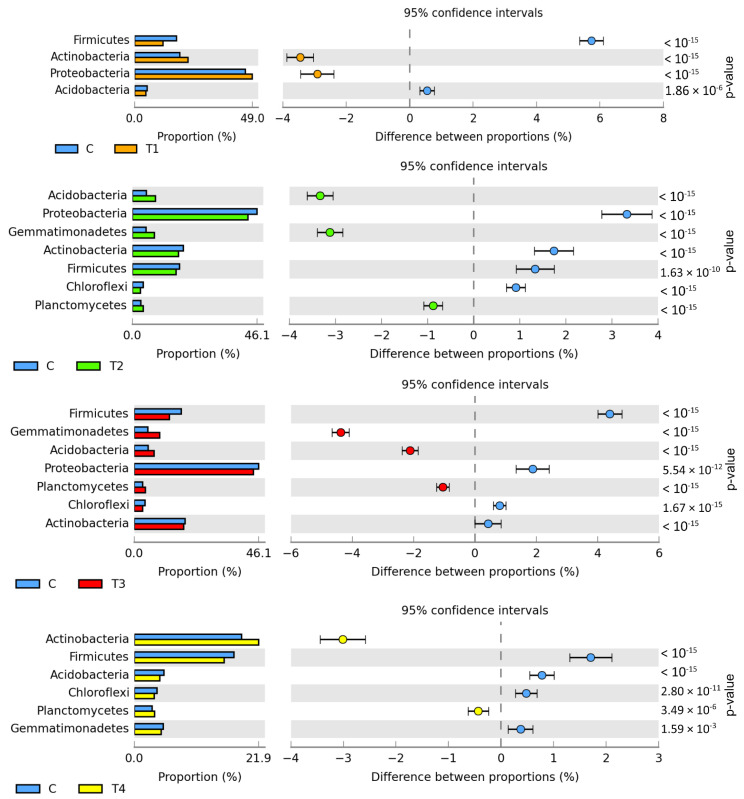
Differences between ratios of bacterial phyla, OTU ≥ 1%. Tebuconazole doses in mg kg^−1^: C—control soil, T1—0.01 mg, T2—0.1 mg, T3—0.5 mg, T4—1.0 mg.

The metagenomic analysis (Figure 1) demonstrated bacteria belonging to *Proteobacteria* to predominate in all soil samples (from 42.81% to 49.05%). Abundant also were *Actinobacteria* (from 17.12% to 22.32%), *Firmicutes* (from 11.78% to 17.52%), *Gemmatimonadetes* (from 4.74% to 9.49%), *Acidobacteria* (from 4.47% to 8.58%), *Planctomycetes* (from 2.99% to 4.18%), and *Chloroflexi* (from 3.06% to 4.01%). Tebuconazole doses of 0.01 and 1.0 mg kg^−1^ caused the relative abundance of *Actinobacteria* to increase by 3.45% and 3.01%, respectively. Moreover, *Proteobacteria* abundance was observed to increase by 2.91% in T1 soil. The relative abundance of *Acidobacteria* and *Gemmatimonadetes* increased in soil samples contaminated with tebuconazole doses of 0.1 and 0.5 mg kg^−1^. The T2 dose increased the abundance of these bacteria by 3.33% and 3.12%, whereas the T3 dose increased by 2.11% and 4.38%, respectively. In turn, a significant reduction was observed in the relative abundance of the *Firmicutes* phylum bacteria in the soil contaminated with tebuconazole doses of T1 (by 5.74%), T2 (by 4.40%), and T3 (by 1.71%). Diminished relative abundance was also observed for *Proteobacteria* in T2 and T3 soil samples (by 3.33% and 1.88%, respectively).

**Figure 2:**
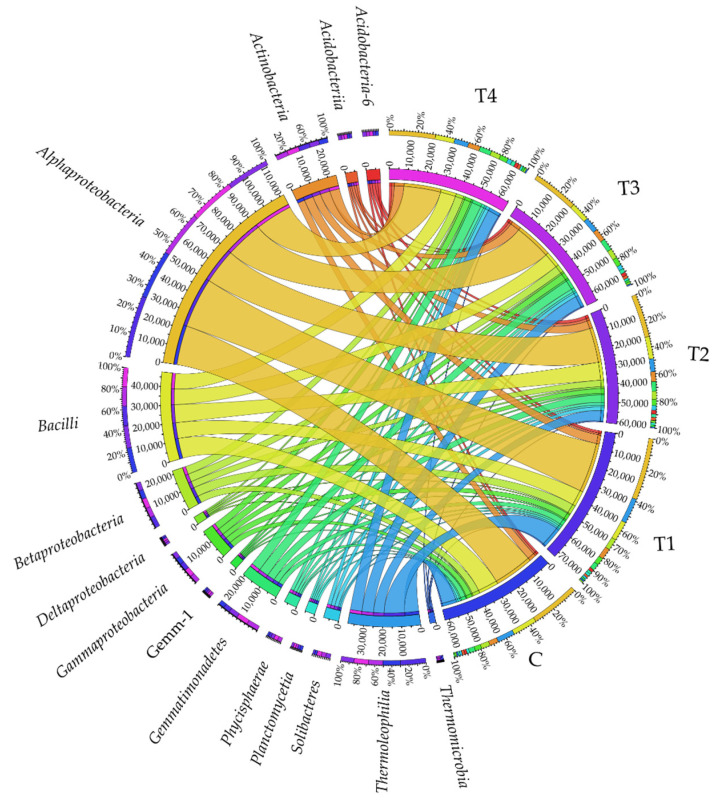
Relative abundance of the predominating bacterial classes in the soils tested with the difference between ratios at ≥1%. Tebuconazole doses in mg kg^−1^: C—control soil, T1—0.01 mg, T2—0.1 mg, T3—0.5 mg, T4—1.0 mg.

At the class taxonomic level, the soils were most densely populated by *Alphaproteobacteria* (Figure 2), with T1 soil found to be the most beneficial for this bacterial class. Another class in terms of abundance turned out to be *Bacilli*, which was definitely the most abundant in C soil, while the least numerous in T1, T3, and T4 soil samples. The *Thermoleophilia* bacteria represented the third class in terms of abundance. They prevailed in T1 soil, which indicated that tebuconazole applied in the T1 dose caused a significant increase in their abundance, and that its remaining doses (T2, T3, and T4) inhibited their development. Tebuconazole administered to the soil in T1–T4 doses had a positive effect on bacteria from the following classes: *Actinobacteria, Gemmatimonadetes*, *Acidobacteria-6*, *Phycisphaerae*, and *Gemm-1*; when applied in T1, T3, and T4 doses on *Betaproteobacteria* and *Gammaproteobacteria*; when applied in T2, T3, and T4 doses on *Solibacteres, Deltaproteobacteria*, and *Acidobacteriia*. At the same time, a tendency was observed for diminishing abundance of *Planctomycetia* and *Thermomicrobia* in response to soil contamination with the tested fungicide.

**Figure 3:**
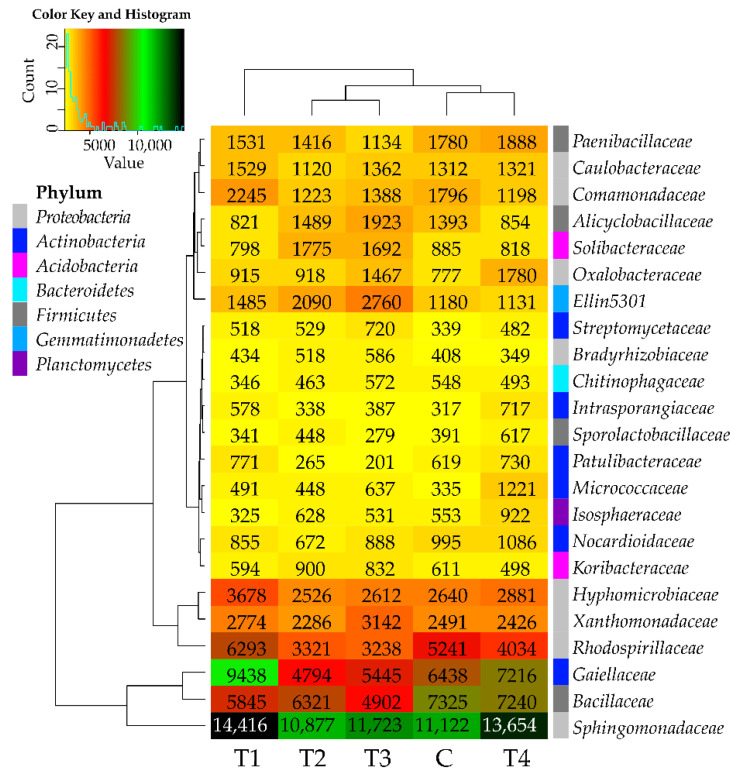
Relative abundance of the predominating bacterial families in the soils tested with the difference between ratios at ≥1%. Tebuconazole doses in mg kg^−1^: C—control soil, T1—0.01 mg, T2—0.1 mg, T3—0.5 mg, T4—1.0 mg.

In all soil samples, the most promoted development was observed in the case of the *Sphingomonadaceae* family bacteria, as their abundance exceeded 10,000 OTUs (Figure 3). Tebuconazole had no significant adverse effect on the bacteria from this family. The abundance ranging from 5000 to 10,000 OTUs was noted for the bacteria from the following families: *Gaiellaceae*, *Bacillaceae*, and *Rhodospirillaceae*. The abundance of the *Bacillaceae* family bacteria was adversely affected by tebuconazole, regardless of its dose, whereas that of *Gaiellaceae*, *Bacillaceae*, and *Rhodospirillaceae* was positively influenced by T1 dose and negatively influenced by T2–T4 doses. Among the families with abundance below 5000 OTUs, tebuconazole was observed to stimulate the development of *Oxalobacteraceae, Micrococcaceae, Intrasporangiaceae*, and *Streptomycetaceae* bacteria. The effects of the tested preparation on the bacteria from the remaining analyzed families were inexplicit. To summarize considerations over tebuconazole effects on bacterial families, it may be concluded that it had no adverse impact on this taxon. The above finding is corroborated by the arrangement of links presented on the dendrogram attached to the heat map, which situate the C and T4 samples in the same group.

**Figure 4:**
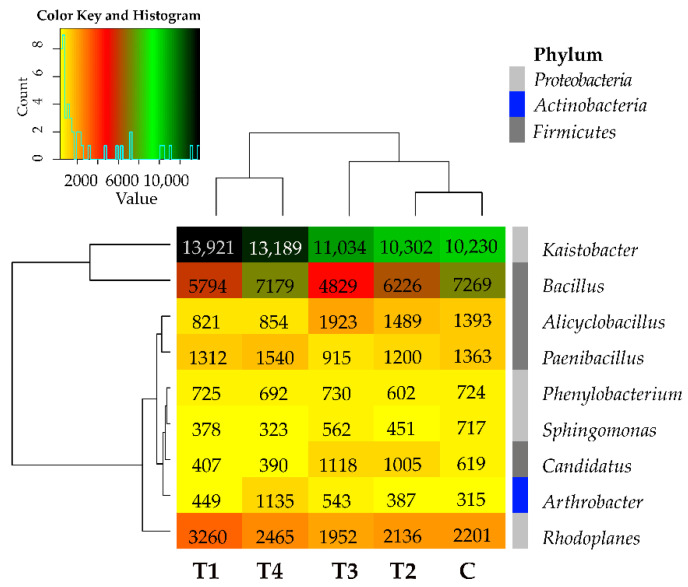
Relative abundance of the predominating bacterial genera in the soils tested with the difference between ratios at ≥1%. Tebuconazole doses in mg kg^−1^: C—control soil, T1—0.01 mg, T2—0.1 mg, T3—0.5 mg, T4—1.0 mg.

Tebuconazole caused no explicit changes in the bacterial structure also at the genus level, as it increased the abundance of the bacteria from *Kaistobacter, Arthrobacter*, and *Streptomyces* genera and diminished the abundance of the bacteria from *Bacillus, Sphingomonas*, and *Gemmata* genera (Figure 4). Its effects varied in the case of the remaining bacterial genera. Worthy of notice is the genus *Kaistobacter* (phylum *Proteobacteria*), whose abundance exceeded 10,000 OTUs in all soil samples. Considering the mean OTU number counted from all soil samples, the additional abundant representative (>1000 OTUs) of the phylum *Proteobacteria* turned out to be the genus *Rhodoplanes*, whereas the additional abundant genera of the phylum *Firmicutes* included *Bacillus, Alicyclobacillus*, and *Peanibacillus*, regardless of tebuconazole dose.

**Figure 5:**
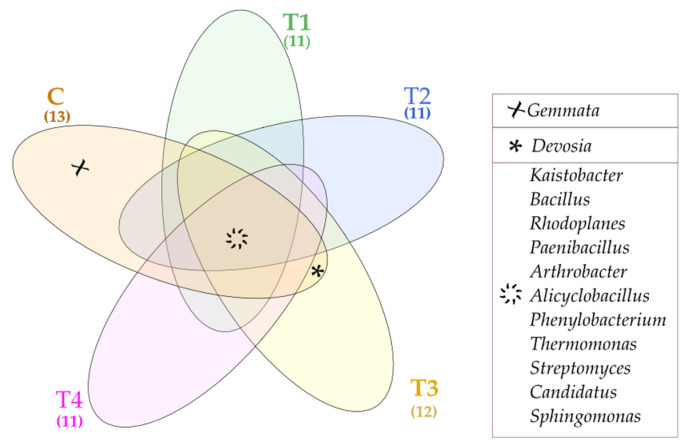
Venne’s diagram depicting unique and common bacterial genera, OTU ≥ 1%. Tebuconazole doses in mg kg^−1^: C—control soil, T1—0.01 mg, T2—0.1 mg, T3—0.5 mg, T4—1.0 mg.

The Venne’s diagram presents unique and common bacterial genera found in particular soil samples (Figure 5). The common genera included: *Kaistobacter*, *Bacillus*, *Rhodoplanes*, *Paenibacillus*, *Arthrobacter*, *Alicyclobacillus*, *Phenylobacterium*, *Thermomonas*, *Streptomyces*, *Candidatus*, and *Sphingomaonas*. In contrast, *Gemmata* turned out to be a unique genus, populating only the control soil (C). The lack of unique bacterial genera in T1–T4 soil samples proves that tebuconazole is safe for the soil environment. 

The values of the Shannon–Wiener (H′) and Simpson (D) indices prove that the bacterial diversity determined at all taxonomic levels was not high in all soil samples (Table S2). In addition, tebuconazole caused no negative changes in the genetic diversity of bacteria, and even increased their diversity at the phylum, class, order, and family levels when administered to the soil in T2–T3 doses. The greatest diversity was noted in the order taxon, whereas the smallest one in the genus taxon. 

### 2.2. Response of Soil Enzymes to Tebuconazole 

The study results demonstrate the positive effects of tebuconazole on the biochemical properties of the soil (Table 3). It stimulated activities of dehydrogenases, alkaline phosphatase, acid phosphatase, arylsulfatase, and β-glucosidase, and did not cause any significant changes in the activities of catalase and urease. In addition, the study showed an increase in the value of the biochemical index of soil quality (BA) upon soil treatment with tebuconazole doses of T1–T4, which points to its positive effect on biochemical processes in the soil environment.

## 3. Discussion

The penetration of fungicides to the soil environment poses a severe threat to non-target organisms, including microorganisms that colonize soil ecosystems. In most cases, soil environment contamination with these chemical substances is due their incorrect application [25]. In the present study, tebuconazole administered to the soil created favorable conditions for the proliferation of organotrophic bacteria and fungi, which could use it as a substrate for their growth and development [26]. In turn, actinobacteria were found to be less tolerant to tebuconazole. Its adverse effect on this bacterial consortium might result from damage caused to their cellular membranes [27], which in turn could lead to disorders in the structure and functioning of their communities. Disorders in the cells of these microorganisms could be triggered by tebuconazole effect on the biosynthesis of amino acids and proteins [27,28]. A study conducted by Muñoz-Leoz et al. [28] demonstrated the potential adverse effect of tebuconazole on soil microorganisms. Its dose of 500 mg kg^−1^ applied to the soil caused microorganism biomass to decrease by as much as 94.6% compared to the control soil. Other research carried out by Wang et al. [29] on samples of river-water soil treated with tebuconazole (doses of 1.0, 10.0, and 100 mg kg^−1^) proved that when applied in the highest dose tested, tebuconazole exerted a negative impact on fungi population. In turn Cycoń et al. [30], who treated soil with tebuconazole doses of 2.7, 13.5, and 270 mg kg^−1^ soil, noted a negligible adverse effect of only the highest tested dose on the biomass of microorganisms. This finding was not corroborated by Dealtry et al. [31], who demonstrated intensive proliferation of microorganisms, including i.a. actinobacteria, in the soil containing tebuconazole. A study conducted by Strickland et al. [32] on sandy-loamy soil proved that tebuconazole used in the field doses had no significant effect on the biomass of soil microorganisms. The present study demonstrated that tebuconazole contributed to increased values of the CD index of the analyzed microorganisms, and that it had various effects on the values of the EP index. Among other things, it increased the EP value of organo-trophic bacteria and actinobacteria and decreased the EP value of fungi. The study also showed that organotrophic bacteria and fungi belonged to r-strategists, therefore their sensitivity to tebuconazole could be greater than that of K-strategists, characterized by greater resistance to changes in environmental conditions [33,34]. Fungicides not only cause changes in the number and activity of microorganisms, but also affect their structure. Therefore, changes taking place in the soil under the influence of fungicides are very well depicted by the structure of microbial communities [12]. Metagenomic analysis enabled observing modifications in the structure of microorganisms as affected by soil contamination with tebuconazole. Storck et al. [35], who evaluated tebuconazole effect on the diversity of structure of bacterial communities, demonstrated that both the control soil and the soil treated with tebuconazole were the most densely colonized by bacteria belonging to *Proteobacteria, Acidobacteria, Bacteroidetes*, and *Actinobacteria*. In the present study, soil treatment with tebuconazole dose T1 promoted the proliferation of *Proteobacteria*. Moreover, study results reported by Wu et al. [36] confirmed the increased relative abundance of *Proteobacteria* in the soil exposed to tebuconazole. The above finding proves the adaptive capabilities of these bacteria to changes triggered by this chemical compound in the environmental conditions [37]. The physicochemical properties of the soil, which may change under the influence of various factors such as how the soil is used, play a significant role in shaping the diversity and structure of bacterial communities. Arunrat et al. [38], while assessing the influence of various farming systems (rice-fish, co-culture and rice monoculture farming system) on the structure of bacterial communities, noted that in both systems the dominant taxa were *Actinobacteria*, *Chloroflexi*, *Proteobacteria*, *Acidobacteria* and *Planctomycetes*. However, the bacterial composition of a rice-fish co-culture system was determined by the soil pH, the content of the clay fraction and the content of the total nitrogen, while in the rice monoculture system by the content of magnesium and sand fraction. In turn, Viruel et al. [39] in Argentina’s semi-arid Chaco ecoregion (which has been converted from pasture to cropland), identified the effects of land uses and management practices (i.e., ungrazed pasture, grazed pasture and cropping systems under zero and conventional tillage) on soil bacterial communities’ structure. The authors noted that the soils were dominated by bacteria belonging to the types *Firmicutes*, *Proteobacteria* and *Actinobacteria*.

Han et al. [40] reported that bacteria from the following genera: *Methylobacterium, Burkholderia, Hyphomicrobium*, and *Dermacoccus*, exhibited a vast potential for tebuconazole degradation, and that *Methylobacterium* bacteria were highly sensitive to its effects. In the present study, tebuconazole increased the relative OTU number of bacteria from *Kaistobacter, Arthrobacter*, and *Streptomyces* genera, and reduced the abundance of these from *Bacillus, Sphingomonas*, and *Gemmata* genera, which may point to their sensitivity to this chemical. In addition, soil contaminated with fungicides has been reported to offer favorable conditions for the development of *Rhodococcus* [41]. 

The effect of fungicides on the soil ecosystem entails the response of not only microorganisms but also enzymes being important biological indicators of soil [8,26,42,43]. The present study demonstrated that tebuconazole stimulated activities of dehydrogenases, alkaline phosphatase, acid phosphatase, arylsulfatase, and β-glucosidase, and caused no significant changes in the activities of catalase and urease. The enhanced enzymatic activity may be due to the increased population of organotrophic bacteria that use tebuconazole as a source of carbon and energy [42]. Anuradha et al. [44] observed enhanced activities of urease and phosphatases upon soil treatment with tebuconazole doses ranging from 1.0 kg ha^−1^ to 5.0 kg ha^−1^. However, its higher doses (7.5 kg ha^−1^ and 10.0 kg ha^−1^) were found to inhibit activities of these enzymes. 

Nevertheless, it is believed that—when applied incorrectly—most fungicides may lead to disorders in the metabolism of microorganisms, which is in turn reflected in the activities of soil enzymes [45,46]. In addition, the suppressed activity of certain soil enzymes may be due to their immobilization by soil colloids or to a small amount of organic matter in the soil [38,47]. The adverse effect of tebuconazole applied in doses of 5.0, 50.0, and 500 mg kg^−1^ on the activities of urease, alkaline phosphatase, β-glucosidase, and arylsulfatase was reported by Muñoz-Leoz et al. [28]. The present study showed that tebuconazole administered to the soil in doses ranging from 0.01 mg kg^−1^ to 1.0 mg kg^−1^ not only had no adverse effects on the activities of soil enzymes, but even activated them.

## 4. Materials and Methods

### 4.1. Tebuconazole 

The study was conducted with tebuconazole with 99.80% purity purchased at Sigma-Aldrich (Taukirchen, Germany). Table 4 presents its selected physicochemical properties.

### 4.2. Soil 

The soil material used in the study derived from the Teaching and Experimental Station in Tomaszkowo village, located in north-eastern Poland, Central Europe (53,7161° N, 20,4167° E). The soil material for the research was collected from the topsoil layer of an arable at a depth of 0–20 cm after spring barley harvested. The soil was classified as Eutric Cambisols [48]. Considering its fraction size composition, it was sandy loam (sand fraction—69.41%, silt fraction—27.71%, and clay fraction—2.88%). It had the following properties: pH_KCl_—7.0, hydrolytic acidity—6.40 mmol^+^ kg^−1^, sum of exchangeable base cations—165.90 mmol^+^ kg^−1^, total exchangeable capacity—172.30 mmol^+^ kg^−1^, degree of saturation of the sorptive complex with base cations—96.28%, total organic carbon content—14.30 g kg^−1^, and total nitrogen content—0.98 g kg^−1^.

### 4.3. Experimental Design

The experiment was carried out under strictly controlled conditions in 3 replications. The soil material (100 g) was placed in glass beakers (150 cm^3^) and treated with the following various doses of tebuconazole (administered in single doses in the form an aquatic emulsion, in mg kg^−1^ soil d.m.: 0.00 (C), 0.01 (T1), 0.10 (T2), 0.50 (T3), and 1.00 (T4). Because studies reported in literature [28,29,30,49,50] regarding the tebuconazole effect on the biological activity of soil have usually focused on its large doses, unseen in agricultural production, the present study aimed to analyze the effect of this chemical added to soil in doses most commonly applied in agricultural practice. After tebuconazole addition, the soil was thoroughly homogenized and moistened to 50% of its capillary water capacity using sterile deionized water, and this moisture content of the soil was maintained throughout the study period. The soil samples were incubated at a temperature of 25 °C for 30 days because the greatest changes caused in soil microbiome by pesticides are usually observed within a month [44,47,51]. Within 30 days of the experiment, the fresh soil (sieved through a screen with 2 mm mesh diameter) was subjected to microbiological and enzymatic analyses.

### 4.4. Microbiological Analyses of Soil

Microbiological analyses of soil were performed with a standard method and with the Next-Generation Sequencing (NGS) method. The population numbers of organotrophic bacteria (Org), actinobacteria (Act), and fungi (Fun) were determined with the serial dilution method in 4 replications. Microorganisms were cultured in an incubator at a temperature of 28 °C for 10 days. During 10-day incubation, grown colonies of microorganisms were counted every day and then the number of their colony forming units (cfu) was determined. The detailed methodology of microbiological analyses and the composition of the media are described in the work by Borowik et al. [52].

Genomic DNA of the bacteria was isolated from the soil by means of a Genomic Mini AX Bacteria + kit (A&A Biotechnology, Gdansk, Poland), using lyticase. The mechanical lysis of the samples was performed with a FastPrep-24 type (MP Biomedicals, Santa Ana, CA, USA) device using zirconia beads. The isolated bacterial DNA was additionally purified by means of an Anti-Inhibitor Kit (A&A Biotechnology, Gdansk, Poland). The presence of bacterial DNA in the tested samples was confirmed in the Real-Time PCR performed in a CFX Connect thermocycler (Bio-rad, Twinsburg, USA), using a SYBR Green dye as fluorochrome. The reaction was performed using universal primers: 1055F (5′-ATGGCTGTCGTCAGCT-3′) and 139R (5′-ACGGGCGGTGTGTAC-3′), amplifying the fragment of a bacterial 16S rRNA gene [53].

The sequencing of bacterial amplicons was conducted with an Illumina MiSeq PE300 (Illumina Inc., San Diego, CA, USA) device in a 2 × 300 bp paired-end mode by Genomed S.A. company (Warsaw, Poland) based on the V3-V4 region of the 16S rRNA gene. The hypervariable region was amplified using specific primers: 341F (5′-CCTACGGGNGGCWGCAG-3′) and 785R (5′-GACTACHVGGGTATCTAATCC-3′). The manuscript presents OTU ≥ 1% data of the obtained bacterial sequences.

### 4.5. Biochemical Analyses of Soil

The soil samples were analyzed for the activities of dehydrogenases (Deh), catalase (Cat) as well as urease (Ure), alkaline phosphatase (Pal), acid phosphatase (Pac), arylsulfatase (Aryl), and β-glucosidase (Glu). Enzymatic activity was determined using the following reagents: dehydrogenases—3% aqueous solution of 2,3,5-triphenyl tetrazolium chloride; catalase—0.3% hydrogen peroxide; urease—10% aqueous solution of urea; alkaline phosphatase and acid phosphatase—0.115 M disodium 4-nitrophenyl phosphate; arylsulfatase—0.02 M potassium-4-nitrophenylsulfate; and β-glucosidase—0.025 M 4-nitrophenyl-β-d-glucopyranoside. Activities of the analyzed enzymes were expressed in the following units: dehydrogenases in µmol TFF kg^−1^ d.m. h^−1^; catalase—mol O_2_ kg^−1^ d.m. h^−1^; urease—mmol N-NH_4_ kg^−1^ d.m. h^−1^; as well as alkaline phosphatase, acid phosphatase, arylsulfatase, and β-glucosidase—mmol PNP kg^−1^ d.m. h^−1^. The procedure for the determination of soil enzymatic activity was presented in the study by Borowik et al. [52].

### 4.6. Physicochemical Analyses of Soil

Before the physicochemical analyses, the soil was air-dried and sieved through a screen with 2 mm mesh diameter. The fraction size composition of the soil was determined using a Mastersizer 2000 laser diffraction particle size analyzer (Malvern, Worcestershire, UK), soil pH—potentiometrically in 1 mol dm^−3^ KCl, hydrolytic acidity and sum of exchangeable base cations—with the Kappen method, organic carbon content—with the Tiurin method, and total nitrogen content—with the Kjeldahl method [54]. 

### 4.7. Bioinformatic and Statistical Computations and Analyses of Study Results

The determined population numbers of organotrophic bacteria, actinobacteria, and fungi were used to compute the colony development index (CD) [55], the ecophysiological diversity index (EP) [33], and the index of microbial abundance growth in specified time intervals (K_s_) [56]. In turn, the number of operational taxonomic units (OTU) of bacteria was used to compute values of the Shannon-Wiener index (H’) and the Simpson index (D) [57]. The determined activities of soil enzymes (Deh, Cat, Ure, Pal, Pac, Aryl, Glu) allowed computing the biochemical index of soil quality (BA) developed by Wyszkowska et al. [58]. The results of the metagenomic analysis were subjected to bioinformatic analysis using the QIIME (Quantitative Insights Into Microbial Ecology) software based on a reference data base GreenGenes v13_8. Bacterial phyla were compared by means of the G test G (w/Yates’) + Fisher test using the STAMP 2.1.3 software [59]. Bacterial classes were presented in the form of a circle using the Circos 0.68 package [60]. Bacterial families and genera were presented in the form of a heat map prepared using the RStudio v1.2.5033 software [61], gplots library [62], and v3.6.2 system [63]. The results of the abundance of microorganisms and activities of soil enzymes were developed statistically using Statistica 13.3 package [64]. Homogenous groups were determined deploying one-way analysis of variance (ANOVA) at *p* = 0.01, by means of the Tukey test. Simple Pearson’s correlation coefficients and standard deviations were computed as well. Unique and common bacterial genera were presented in the form of a Venne’s diagram using the InteractiVenn software [65].

## 5. Conclusions

The conducted study provided valuable information about the response of soil microorganisms and enzymes to tebuconazole administered to the soil, which when used in field doses did not diminish its fertility. It stimulated the proliferation of organotrophic bacteria and fungi as well as activities of most of the analyzed soil enzymes, causing no significant changes in the structure of bacterial communities. All analyzed soil samples were most densely populated by *Proteobacteria* bacteria, but relatively high abundance was also noted for *Actinobacteria*, *Firmicutes*, *Gemmatimonadetes*, *Acidobacteria*, *Planctomycetes*, and *Chloroflexi*. The most abundant bacteria in the soil samples treated with tebuconazole were these belonging to the following genera: *Kaistobacter*, *Arthrobacter*, and *Streptomyces*, which proves that these bacteria should be perceived as potential candidates for an effective vaccine for the bioaugmentation of soil contaminated with the fungicide. 

## Figures and Tables

**Table 1:** Effect of tebuconazole on the colony development index (CD) of microorganisms.

Object	Organotrophic Bacteria	Actinobacteria	Fungi
C	43.086 ± 0.908 ^c^	20.290 ± 0.331 ^d^	30.119 ± 0.919 ^d^
T1	43.357 ± 0.256 ^c^	47.087 ± 1.213 ^a^	33.989 ± 0.433 ^c^
T2	55.236 ± 1.115 ^a^	24.177 ± 1.804 ^c^	37.711 ± 0.358 ^b^
T3	53.462 ± 0.525 ^a^	28.071 ± 0.701 ^b^	37.697 ± 0.500 ^b^
T4	47.123 ± 0.518 ^b^	23.091 ± 0.295 ^cd^	42.059 ± 0.498 ^a^
$\bar{x}$	48.453	28.543	36.315
*r*	0.185	−0.321	0.847 *

Tebuconazole doses in mg kg^−1^: C—control soil, T1—0.01 mg, T2—0.1 mg, T3—0.5 mg, T4—1.0 mg; *r*—simple Pearson’s correlation coefficient significant at * *p* < 0.05, n = 20; $\bar{x}$—arithmetic mean; ±—standard deviation. Homogeneous groups designated with the same letters (^a–d^) were calculated separately for each group of microorganisms.

**Table 2:** Effect of tebuconazole on the ecophysiological diversity of soil microorganisms measured using the EP index.

Object	Organotrophic Bacteria	Actinobacteria	Fungi
C	0.698 ± 0.015 ^b^	0.861 ± 0.003 ^a^	0.708 ± 0.026 ^a^
T1	0.401 ± 0.019 ^d^	0.658 ± 0.057 ^b^	0.697 ± 0.019 ^a^
T2	0.661 ± 0.011 ^b^	0.861 ± 0.006 ^a^	0.700 ± 0.011 ^a^
T3	0.596 ± 0.015 ^c^	0.834 ± 0.023 ^a^	0.565 ± 0.026 ^b^
T4	0.782 ± 0.005 ^a^	0.853 ± 0.043 ^a^	0.486 ± 0.014 ^c^
$\bar{x}$	0.628	0.813	0.631
*r*	0.565	0.352	−0.986 *

Tebuconazole doses in mg kg^−1^: C—control soil, T1—0.01 mg, T2—0.1 mg, T3—0.5 mg, T4—1.0 mg; *r*—simple Pearson’s correlation coefficient significant at * *p* < 0.05, n = 20; $\bar{x}$ —arithmetic mean; ±—standard deviation. Homogeneous groups designated with the same letters (^a–d^) were calculated separately for each group of microorganisms.

**Table 3:** Effect of tebuconazole on the activity of soil enzymes in 1 kg soil d.m.

Object	Deh µmol TFF	Cat mol O_2_	Ure mmol N-NH_4_	Pal	Pac	Aryl	Glu	BA
				mmol PNP				
C	1.541 ± 0.075 ^b^	0.071 ± 0.004 ^ab^	0.391 ± 0.029 ^ab^	0.191 ± 0.010 ^b^	1.270 ± 0.082 ^c^	0.039 ± 0.003 ^b^	0.213 ± 0.005 ^b^	3.716 ± 0.221 ^b^
T1	1.967 ± 0.025 ^a^	0.078 ± 0.004 ^a^	0.426 ± 0.030 ^a^	0.202 ± 0.012 ^ab^	1.294 ± 0.024 ^bc^	0.039 ± 0.007 ^b^	0.217 ± 0.004 ^ab^	4.224 ± 0.052 ^a^
T2	1.845 ± 0.034 ^a^	0.066 ± 0.004 ^ab^	0.399 ± 0.030 ^ab^	0.214 ± 0.023 ^ab^	1.425 ± 0.019 ^ab^	0.044 ± 0.004 ^a^	0.230 ± 0.006 ^ab^	4.224 ± 0.026 ^a^
T3	1.849 ± 0.047 ^a^	0.065 ± 0.004 ^ab^	0.390 ± 0.031 ^ab^	0.238 ± 0.013 ^a^	1.474 ± 0.013 ^a^	0.046 ± 0.002 ^a^	0.247 ± 0.019 ^a^	4.309 ± 0.076 ^a^
T4	1.791 ± 0.022 ^ab^	0.058 ± 0.004 ^b^	0.298 ± 0.030 ^b^	0.241 ± 0.005 ^a^	1.455 ± 0.026 ^a^	0.047 ± 0.002 ^a^	0.248 ± 0.003 ^a^	4.138 ± 0.057 ^a^
$\bar{x}$	1.799	0.068	0.381	0.217	1.384	0.043	0.231	4.122
*r*	0.088	−0.855 *	−0.908 *	0.892 *	0.734	0.848 *	0.881 *	0.297

Deh—dehydrogenases, Cat—catalase, Ure—urease, Pal—alkaline phosphatase, Pac—acid phosphatase, Aryl—arylsulfatase, Glu—β-glucosidase, BA—biochemical index of soil quality. Tebuconazole doses in mg kg^−1^: C—control soil, T1—0.01 mg, T2—0.1 mg, T3—0.5 mg, T4—1.0 mg; *r*—simple Pearson’s correlation coefficient significant at * *p* < 0.05, n = 15; $\bar{x}$ —arithmetic mean; ±—standard deviation. Homogeneous groups designated with the same letters (^a–c^) were calculated separately for each group of enzymes.

**Table 4:** Selected physicochemical properties of tebuconazole [47].

Parameter (Temperature 20–25 °C, pH—7)	Formula/Value
Chemical formula	C_16_H_22_CIN_3_O
Solubility in water (mg dm^−3^)	36
Solubility in organic solvents at 20 °C (mg dm⁻^3^)	200,000
log K_ow_	3.70
p*K_a_*	5.0
Vapor pressure (mPa)	1.3 × 10^−3^
Aqueous hydrolysis	stable
K*_f_* (cm^3^ g^−1^)	12.69
K*_foc_* (cm^3^ g^−1^)	769
DT_50_ (days)	63
EC_50_ (mg dm^−3^)	2.79

## Data Availability

Not applicable.

## Supplementary Materials

The following supporting information can be downloaded at: https://www.mdpi.com/article/10.3390/molecules27217501/s1, Figure S1: Increase in the abundance of microorganisms in various time intervals, in % (Ks); Table S1: Effect of tebuconazole on population numbers of microorganisms, 10^n^ cfu kg^−1^ soil d.m.; Table S2: Values of Shannon–Wiener index and Simpson index computed based on all OTU data.

## Sample Availability

Samples of the compound are not available from the authors.
