# Supplementary material for: Effects of Tebuconazole Application on Soil Microbiota and Enzymes
Source: Molecules. 2022 Nov 3;27(21):7501. doi: 10.3390/molecules27217501 (PMC9656111; doi:10.3390/molecules27217501)
Supplement: Supplementary file 1 [file molecules-27-07501-s001.zip › molecules-1984162-supplementary.pdf]

Supplementary materials

Effects of tebuconazole application on soil microbiota and enzymes

Małgorzata Baćmaga [1], Jadwiga Wyszkowska [1,*], Agata Borowik [1] and Jan Kucharski [1]

[1] Department of Soil Science and Microbiology; Faculty of Agriculture and Forestry; University of Warmia and Mazury in Olsztyn, Plac Łódzki 3, 10-727 Olsztyn, Poland
* Correspondence: jadwiga.wyszkowska@uwm.edu.pl

Table S1. Effect of tebuconazole on population numbers of microorganisms, 10^n cfu kg^{-1} soil d.m.

Object	Organotrophic bacteria 10^{10} cfu	Actinobacteria 10^{10} cfu	Fungi 10^8 cfu
C	1.599±0.077[e]	0.731±0.019[b]	0.175±0.008[e]
T1	1.670±0.006[d]	0.987±0.011[a]	0.327±0.006[d]
T2	2.052±0.017[c]	0.690±0.030[c]	0.375±0.018[c]
T3	2.242±0.018[b]	0.630±0.027[cd]	0.479±0.003[b]
T4	2.575±0.024[a]	0.630±0.028[cd]	0.606±0.012[a]
$\overline{x}$	2.028	0.734	0.392
r	0.936*	-0.617	0.912

Tebuconazole doses in mg kg^{-1}: C – control soil, T1 – 0.01 mg, T2 – 0.1 mg, T3 – 0.5 mg, T4 – 1.0 mg; *r* – simple Pearson's correlation coefficient significant at *$p<0.05$, n=20; $\overline{x}$ – arithmetic mean; ± – standard deviation. Homogeneous groups designated with the same letters (a-e) were calculated separately for each group of microorganisms.

Table S2. Values of Shannon-Wiener index and Simpson index computed based on all OTU data.

Object	Taxa				
	Phylum	Class	Order	Family	Genus
	Shannon-Wiener index				
C	1.718±0.006^{b}	2.419±0.007^{b}	2.928±0.005^{b}	3.025±0.006^{c}	1.835±0.012^{a}
T1	1.641±0.014^{d}	2.377±0.004^{c}	2.933±0.005^{b}	2.973±0.005^{d}	1.715±0.007^{b}
T2	1.802±0.004^{a}	2.552±0.008^{a}	3.012±0.007^{a}	3.063±0.006^{b}	1.825±0.006^{a}
T3	1.788±0.009^{a}	2.566±0.003^{a}	3.032±0.005^{a}	3.106±0.003^{a}	1.851±0.013^{a}
T4	1.690±0.007^{c}	2.419±0.006^{b}	2.902±0.006^{c}	3.034±0.008^{c}	1.828±0.011^{a}
$\bar{x}$	1.728	2.467	2.961	3.040	1.811
r	0.213	0.347	0.130	0.486	0.354
	Simpson index				
C	0.737±0.006^{ab}	0.688±0.006^{bc}	0.748±0.010^{c}	0.655±0.006^{a}	0.355±0.016^{a}
T1	0.710±0.008^{b}	0.661±0.010^{c}	0.798±0.007^{a}	0.610±0.006^{b}	0.263±0.009^{c}
T2	0.764±0.006^{a}	0.718±0.013^{a}	0.763±0.006^{bc}	0.628±0.010^{ab}	0.330±0.007^{a}
T3	0.757±0.004^{a}	0.716±0.009^{a}	0.794±0.007^{ab}	0.623±0.008^{b}	0.298±0.012^{b}
T4	0.731±0.009^{ab}	0.700±0.008^{ab}	0.764±0.006^{bc}	0.634±0.005^{ab}	0.347±0.008^{a}
$\bar{x}$	0.740	0.697	0.773	0.630	0.319
r	0.257	0.534	0.205	-0.277	0.079

Tebuconazole doses in mg kg^{-1}: C – control soil, T1 – 0.01 mg, T2 – 0.1 mg, T3 – 0.5 mg, T4 – 1.0 mg; *r* – simple Pearson's correlation coefficient significant at **p*<0.05, n=20; $\bar{x}$ – arithmetic mean; ± – standard deviation. Homogeneous groups designated with the same letters (a-d) were calculated separately for each group of microorganisms.

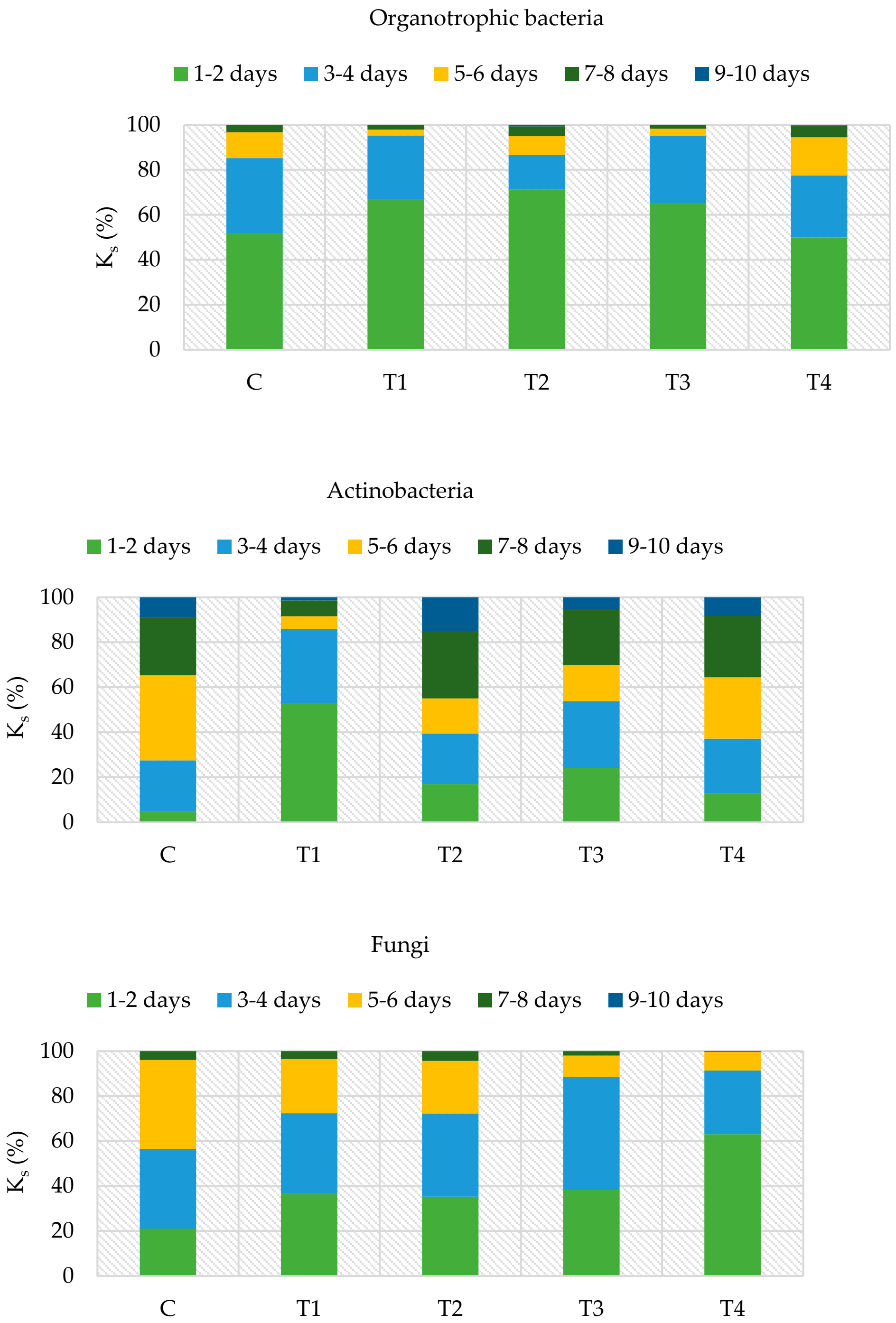

Figure S1. Increase in the abundance of microorganisms in various time intervals, in % (K_s). Tebuconazole doses in mg kg^{-1}: C – control soil, T1 – 0.01 mg, T2 – 0.1 mg, T3 – 0.5 mg, T4 – 1.0 mg.
